# Metagenomics-based systematic analysis reveals that gut microbiota Gd-IgA1-associated enzymes may play a key role in IgA nephropathy

**DOI:** 10.3389/fmolb.2022.970723

**Published:** 2022-08-24

**Authors:** Xiaolin Liang, Simeng Zhang, Difei Zhang, Liang Hu, La Zhang, Yu Peng, Yuan Xu, Haijing Hou, Chuan Zou, Xusheng Liu, Yang Chen, Fuhua Lu

**Affiliations:** ^1^ The Second Clinical College of Guangzhou University of Chinese Medicine, Guangzhou, China; ^2^ Department of Nephrology, Guangdong Provincial Hospital of Chinese Medicine, Guangzhou, China; ^3^ Guangdong-Hong Kong-Macau Joint Lab on Chinese Medicine and Immune Disease Research, Guangzhou University of Chinese Medicine, Guangzhou, China; ^4^ Hunan Academy of Traditional Chinese Medicine Affiliated Hospital, Changsha, China

**Keywords:** immunoglobulin a nephropathy, gut microbiota, galactose-deficient IgA1, *Flavonifractor plautii*, α-galactosidase, α-N-acetyl-galactosaminidase, metagenomics sequencing.

## Abstract

**Background:** IgA nephropathy (IgAN) is the most common type of glomerulonephritis in Asia. Its pathogenesis involves higher expression of galactose-deficient IgA1 (Gd-IgA1) and dysregulated intestinal mucosal immunity. The objective of this study was to explore whether specific gut microbiota and associated enzymes affect Gd-IgA1 in IgAN.

**Methods:** This study carried out shotgun metagenomic sequencing with Illumina on fecal samples collected from 20 IgAN patients (IgAN group) and 20 healthy controls (HCs group) who were recruited from January 2016 to December 2018 at the Second Clinical College of Guangzhou University of Chinese Medicine. Differences analysis in gut microbiota was performed to determine the overall microbiota composition, the representative enterotypes, and the microbiota abundance. Correlations between gut microbiota and clinical indicators were assessed by Spearman’s analysis. Moreover, the functional prediction of microbial communities and the quantitative calculation of enzymes encoded by microbiome were performed using the MetaCyc pathway and the bioBakery three platform, respectively.

**Results:** *Bacteroides plebeius* and *Bacteroides vulgatus* levels were higher, while *Prevotella copri* and *Alistipes putredinis* levels were lower in the IgAN group compared to HCs group. Enterotype I characterized by *Bacteroides* was closely related to the IgAN patients. Moreover, *Bacteroides fragilis*, *Flavonifractor plautii* and *Ruminococcus gnavus* were characteristic bacteria enriched in IgAN patients. Spearman’s correlation analysis found that *Eggerthella lenta* and *Ruminococcus bromii* were positively correlated with urine protein-creatinine ratio, while *Ruminococcus gnavus* showed a direct association with red blood cells in urine, and *Bacteroides vulgatus* and *Ruminococcus gnavus* were positively correlated with eGFR. These results indicated that intestinal dysbacteriosis occurred in IgAN patients and was associated with clinical and biochemical features. In addition, MetaCyc pathway analysis predicted microbiota-related metabolic pathways, including the biosynthesis of amino acids and glycans, were associated with the IgAN group. Microbial enzymes analysis highlighted that Gd-IgA1-associated α-galactosidase and α-N-acetyl-galactosaminidase secreted by *Flavonifractor plautii* were enriched in IgAN patients.

**Conclusion:** These findings suggested that α-galactosidase and α-N-acetyl-galactosaminidase secreted by *Flavonifractor plautii* might be related to the production of Gd-IgA1, indicating that enzymes originated from abnormal intestinal microbiota may contribute to the production of Gd-IgA1 and play an important role in the pathogenesis of IgAN.

## Introduction

Immunoglobulin A nephropathy (IgAN) is the most common form of primary glomerulonephritis (Wyatt and Julian, 2013), which is the second leading cause of chronic kidney disease, accounting for 30–50% of patients with end-stage renal disease (ESRD) (Maixnerova et al., 2016). Currently, IgAN is recognized as an immune-mediated disease defined by the deposition of polymeric and hypogalactosylated IgA1 in the glomerular mesangium (Chebotareva et al., 2020). Although the overproduction of aberrantly glycosylated IgA1 (Wyatt and Julian, 2013) is considered to be an indispensable pathogenic factor in IgAN, the origin of galactose-deficient IgA1 (Gd-IgA1) remains unclear. Recently, several studies have suggested that the gut-associated immune system may be involved in the pathogenesis of IgAN (Coppo, 2018; He et al., 2020; Selvaskandan et al., 2022). IgA, the primary antibody isotype observed in mucosal secretions, is playing a vital role in the modification of gut microbiota composition by symbiosis among bacteria (Nakajima et al., 2018). The gut microbiota is essential to maintain intestinal immune homeostasis, but defects in the mucosal microenvironments and imbalances in the gut microbiota might contribute to the pathogenesis of IgAN (Floege and Feehally, 2016). Most strikingly, Zachova et al. (2022) demonstrated that the mucosal microenvironment may play a pivotal role in driving the production of Gd-IgA1. Hence, it is necessary to clarify the dysregulation of IgA from the view of the gut microbiome since it should be of decisive significance for unveiling the origin of Gd-IgA1 in IgAN.

IgAN is characterized by the presence of IgA-dominant or co-dominant immune deposits within glomeruli and its pathogenesis relates to an aberrant form of IgA1 (Lai, 2012; Roberts, 2014). Unlike other immunoglobulins, human IgA1 has a distinctive hinge region, which is the binding site of three to six O-glycan chains (Lai, 2012). In normal IgA1, these O-glycans are featured by the presence of N-acetylgalactosamine (GalNAc) with a β1,3-linked galactose (Gal) and connected by sialic acid (Ohyama et al., 2021). It is reported that aberrant IgA1 is presented as a galactose-deficient IgA1 with a terminal GalNAc or sialylated GalNAc in IgAN patients (Wada et al., 2010). GalNAc and Gal are both antigen-binding sites for the carbohydrate recognition domains of glycan-specific antibodies, glycan-binding proteins and lectins (Kulkarni et al., 2006). Notably, this specific glycan recognition occurs not only in immune responses mediated by IgA1 and Gd-IgA1 but also in the immune response of ABO blood groups in blood transfusion (Hofmann et al., 2017). Interestingly, the carbohydrate chains that determine the ABO blood group are stereoselective constructs of GalNAc for A antigen and D-galactose for B antigen (Hara et al., 2013), which indicates that GalNAc and Gal glycan modification can distinguish the A, B and O group antigens. A recent study has found that an enzymatic pathway stimulated by α-galactosidase and α-N-acetyl-galactosaminidase from *Flavonifractor plautii*, could remove the GalNAc or Gal structures from A/B antigens, as a way of converting A or B RBCs to O (Rahfeld et al., 2019). Both A/B antigens and pathogenic IgA1 in IgAN patients are equally affected by modifications of N-acetylgalactosamine and galactose. However, few studies have investigated enzymatic reactions associated with aberrant intestinal bacteria that influence the production of Gd-IgA1 in the pathogenesis of IgAN. Thus, we hypothesize that the gut microbiome influences IgAN not only through microecological homeostasis but also through enzymatic reactions motivated by dysregulated intestinal bacteria, both of which may lead to a sustained increase in Gd-IgA1.

In the present study, we investigated the microbial communities in the stool of IgAN patients using metagenomics sequencing and assessed whether enzymatic reactions triggered by dysfunctional intestinal bacteria were associated with the production of Gd-IgA1. We found differences in gut microbiota between IgAN and healthy subjects and underlined that the α-galactosidase and α-N-acetyl-galactosaminidase expressed by *Flavonifractor plautii* were enriched in IgAN patients, which may be associated with the production of Gd-IgA1. These findings indicated that key enzymes derived from the intestinal microbiota may be explored as potential therapeutic targets for IgAN.

## Materials and methods

### Study subjects

In this retrospective study, a total of 20 patients with IgAN, confirmed by biopsy, and 20 healthy controls (HCs) were enrolled between January 2016 and December 2018 at the Guangdong Provincial Hospital of Chinese Medicine, the Second Clinical College of Guangzhou University of Chinese Medicine. The diagnostic criteria for IgAN were based on the KDIGO Clinical Practice Guidelines for Glomerulonephritis. Individuals with secondary IgAN, acute myocardial infarction, stroke, acute gastroenteritis, heart failure, malignancies, and those who had taken antibiotics or probiotics within the last 4 weeks were excluded. In our present study, the origin of healthy controls was from the relatives of IgAN patients. This study was approved by the Ethics Committee of the Guangdong Provincial Hospital of Chinese Medicine (No.YE2018-125-01) and written informed consent was obtained from all participants. Dietary and lifestyle information over the past 3 months was collected through questionnaires, as listed in Supplementary Table S1. Dietary habits, which contains weekly consumption frequency of six categories of food, including refined carbohydrates, red meat, white meat, vegetables, fruits, and probiotic drink, were investigated using food frequency questionnaires (Perez Rodrigo et al., 2015). Dietary frequency was defined as follows: always (everyday), usually (4–6 days per week), sometimes (1–3 days per week), and never (0–1 day per week).

### Baseline and laboratory measurements of IgAN

Age, sex, height, weight, systolic blood pressure, diastolic blood pressure and comorbidities were collected. Body mass index (BMI) and mean arterial pressure (MAP) were calculated. The estimated glomerular filtration rate (eGFR) was calculated by the Chronic Kidney Disease Epidemiology Collaboration (CKD-EPI) equation. Fasting peripheral venous blood samples were drawn in the morning in order to obtain serum biochemical parameters, including: hemoglobin (Hb), total protein (TP), albumin (ALB), globulin (GLB), fasting blood glucose (FBG), serum creatinine (sCr), urea, uric acid (UA), triglyceride (TG), total cholesterol (TC), high-density lipoprotein cholesterol (HDL-C), low-density lipoprotein cholesterol (LDL-C), high-sensitivity C-reactive protein (hsCRP), immunoglobulin A (IgA), immunoglobulin G (IgG), immunoglobulin M (IgM), complement 3 (C3), complement 4 (C4) and total complement activity (CH50). First-morning urine samples were also collected to assess hematuria (U-RBC), 24-hour urine protein (24 h U-PRO), urine protein creatinine ratio (uPCR), urine immunoglobulin kap (kapU), urine immunoglobulin lam (lamU), urine immunoglobulin G (IgGU), urine β2-microglobulin (β2-Mg), albuminuria (ALBU), urine α1 microglobulin (α1-MU), urine α 2 macroglobulin (α2-MU) and urine Transferrin (TrfU). Histological findings were graded according to the Oxford classification (Working Group of the International IgA Nephropathy Network and the Renal Pathology Society et al., 2009a; Working Group of the International IgA Nephropathy Network and the Renal Pathology Society et al., 2009b; Trimarchi et al., 2017). Detailed information is shown in the Supplementary Table S2.

### Metagenomics sequencing and bioinformatics analysis

Fecal samples were collected in sterile plastic tubs and kept at −80°C until DNA extraction. We used Magnetic Soil and Stool DNA Kit (TIANGEN BIOTECH, DP712, BEIJING, China) to extract genome DNA from fecal samples following the instructions and procedures. Metagenomics sequencing was performed for high-quality fecal DNA samples (>3 μg DNA with good integrity and low degradation) using second-generation sequencing.

The extracted DNA was randomly fragmented into approximately 150 bp and these fragments were used to construct the library using TruSeq DNA HT Sample Prep Kit (Illumina). The qualified libraries were selected to sequence the fragments using the Paired-End strategy in the illumina Hiseq X Ten platform. We used WGS libraries for metagenomic survey, and the sequencing data of each feces sample was not less than 10Gb and 80,000,00 Reads. MetaPhlAn2 was used for a computational tool for profiling the composition of microbial communities from metagenomic shotgun sequencing data (Truong et al., 2015). Usage and Principle were as follows. 1) The determination of marker: MetaPhlAn2 relied on∼1 M unique clade-specific marker genes identified from∼17,000 reference genomes (∼13,500 bacterial and archaeal, ∼3,500 viral, and ∼110 eukaryotic); 2) Reads alignment to markers using bowtie2 with the strictest mode: -very-sensitive (-D20 -R3 -N0 -L20 -i S,1,0.50); 3) Quantification: The classifier normalized all the reads in every clade through the nucleotide length of its markers and provided the relative abundance of each taxonomic unit. Finally, microbial clade abundances were thus estimated by normalizing read-based counts by the average genome size of each clade. After removing low-quality and human sequences, we performed metagenomics bioinformatics analysis using the bioBakery three platform (Beghini et al., 2021).

Data for metagenomics sequencing were analyzed using the R software (ver. 3.4.3). The community ecology analysis was performed through the Vegan package, while the differences in the relative abundance of the taxa between groups on bacteria, metabolites, and genes were assessed through non-parametric, relative abundance Kruskal-Wallis and Wilcoxon rank sum tests. Community profiling was employed to detect taxonomic differences from phylum to species in IgAN patients and HCs. The sample size and sequence accuracy were determined by rarefaction curves. Community richness and composition were assessed by calculating α and β diversity from MicrobiomeAnalyst. Principal coordinate analysis (PCoA) was conducted to compare the enterotypes of the samples. Meanwhile, the linear discriminant analysis (LDA) effect size (LEfSe) algorithm was used to assess differential abundance at the phylum to species levels, where LDA score >2.0 and *p*-value <0.05 were considered as statistically significant. The Spearman’s rank correlation coefficient was used to calculate the association of demographic and biochemical data with bacterial species. Detected genes were further regrouped into gene families and pathways and then sum-normalized using HUMAnN2 with UniRef90 database (Franzosa et al., 2018). The Functional annotation of microbial communities was performed using the MetaCyc pathway, while the quantitative calculation of enzymes encoded by microbiome were based on uniref90 level4ec. Factorial analysis and one-way analysis of variance were used to compare differences when data followed a normal distribution. *p* <0.05 was used to infer significant differences.

Fecal metagenomic sequencing reads can be downloaded from CNGB Nucleotide Sequence Archive (http://db.cngb.org/cnsa/) under accession number CNP0003090.

### Statistical analysis

For baseline data and laboratory findings, quantitative data were presented either as medians and interquartile ranges or mean ± standard deviation. Other variables were presented as percentages and compared using Wilcoxon rank-sum test or Fisher’s exact test. Statistical analyses of baseline and biochemical data were performed using GraphPad Prism7 and SPSS software (version 21, IBM SPSS Inc., Chicago, IL, United States).

## Results

### Baseline characteristics of study subjects

In this study, a total of 20 IgAN patients and 20 healthy controls were included. Dietary and lifestyle information were provided in Table 1, and there were no significant differences in lifestyle and dietary habits between the two groups, execpt for the weekly frequency of vegetable intake (*p* = 0.001). Besides, basic characteristics in IgAN patients were showed in Table 2. There were 10 men and 10 women with IgAN, with a mean age of 38.5 years (range, 18–50 years). Regarding clinical indicators, IgAN patients showed a urine protein-creatinine ratio of 1.9 ± 4.4 g/g while there were 95.4 ± 142.5 red blood cells/μl of urine (range, 8–582.8 cells/μl). The mean eGFR of IgAN patients was 84.0 ± 30.0 ml/min/1.73 m^2^.

**TABLE 1:** Frequency list of lifestyle and dietary habits in IgAN patients and healthy controls.

Variables	IgAN (*n* = 20)	HC (*n* = 20)	*p*-Value
Lifestyle			
Actual hours of sleep			0.106
<6h	0	4 (20%)	
6–9h	19 (95%)	15 (75%)	
>9h	1 (5%)	1 (5%)	
Sleep quality			0.365
Excellent	6 (30%)	10 (50%)	
Good	11 (55%)	7 (35%)	
Poor	3 (15%)	3 (15%)	
Dietary habits			
Refined carbohydrates			0.194
Always	14 (70%)	15 (75%)	
Usually	3 (15%)	5 (25%)	
Sometimes	3 (15%)	0	
Never	0	0	
Red meat			0.432
Always	1 (5%)	2 (10%)	
Usually	13 (65%)	8 (40%)	
Sometimes	4 (20%)	5 (25%)	
Never	2 (10%)	5 (25%)	
White meat			0.065
Always	0	6 (30%)	
Usually	10 (50%)	7 (35%)	
Sometimes	7 (35%)	5 (25%)	
Never	3 (15%)	2 (10%)	
Vegetables			0.001
Always	14 (70%)	4 (20%)	
Usually	5 (25%)	16 (80%)	
Sometimes	1 (5%)	0	
Never	0	0	
Fruits			0.371
Always	4 (20%)	7 (35%)	
Usually	7 (35%)	8 (40%)	
Sometimes	8 (40%)	3 (15%)	
Never	1 (5%)	2 (10%)	
Probiotic drink			1.000
Always	0	0	
Usually	2 (10%)	3 (15%)	
Sometimes	7 (35%)	6 (30%)	
Never	11 (55%)	11 (55%)	
Taste preferences			0.693
Bland	12 (60%)	9 (45%)	
Salty	5 (25%)	8 (40%)	
Spicy	3 (15%)	3 (15%)	

Note: The *p* value is significant if <0.05.

Legend: Always = everyday; usually = 4–6 days per week; sometimes = 1–3 days per week; never = 0–1 day per week.

**TABLE 2:** Baseline characteristics of the IgAN patients.

Characteristics	IgAN (*n* = 20)
Mean ± SD or n (%) or n	
Demographoc	
Age (years)	38.5 ± 8.7
Male gender (%)	10(50%)
Height (m)	1.6 ± 0.1
Weight (kg)	57.9 ± 9.9
BMI (kg/m^2^)	22.3 ± 3.7
SBP (mmHg)	125.9 ± 19.4
DBP (mmHg)	79.6 ± 11.9
MAP (mmHg)	95.0 ± 13.7
Smoker (%)	0
Alcohol user (%)	8 (38.10%)
Comorbidities	
Hypertension	5(25%)
Hyperlipemia	6(30%)
Diabetes	0
Hyperuricemia	6(30%)
Chronic Gastritis	1(5%)
Hepatic steatosis	4(20%)
Laboratory values	
Hb (g/L)	131.3 ± 10.7
TP (g/L)	63.9 ± 8.0
ALB (g/L)	38.2 ± 6.5
GLB (g/L)	25.8 ± 3.2
FBG (mmol/L)	5.8 ± 2.2
sCr (μmol/L)	97.0 ± 39.2
Urea (mmol/L)	5.5 ± 1.5
UA (μmol/L)	420.0 ± 76.5
eGFR (ml/min/1.73㎡)	84.0 ± 30.0
TG (mmol/L)	1.8 ± 1.5
TC (mmol/L)	5.4 ± 1.8
HDL-C (mmol/L)	1.2 ± 0.4
LDL-C (mmol/L)	3.7 ± 1.6
hsCRP (mg/L)	1.9 ± 2.4
IgA (g/L)	3.2 ± 1.0
IgG (g/L)	10.2 ± 2.3
IgM (g/L)	1.1 ± 0.6
C3 (g/L)	1.1 ± 0.2
C4 (g/L)	0.2 ± 0.1
CH50 (g/L)	30.2 ± 7.9
U-RBC (cells/μL)	95.4 ± 142.5
24h U-PRO (g/24h)	1.7 ± 2.7
uPCR (g/g)	1.9 ± 4.4
D-RBC/U-RBC	0.8 ± 0.2
kapU (mg/L)	71.0 ± 142.7
lamU (mg/L)	44.4 ± 99.4
IgGU (mg/L)	85.1 ± 86.3
β2-Mg (mg/L)	0.9 ± 1.8
ALBU (mg/L)	2226.1 ± 4784.1
α1-MU (mg/L)	26.2 ± 38.0
α2-MU (mg/L)	4.6 ± 5.2
TrfU (mg/L)	53.6 ± 42.1
Histological findings, n	
M0/M1	1/19
E0/E1	17/3
S0/^1^S	14/6
T0/T1/T2	16/3/1
C0/C1/C2	6/13/1

BMI, body mass index; SBP, systolic blood pressure; DBP, diastolic blood pressure; MAP, mean arterial pressure; Hb, hemoglobin; TP, total protein; ALB, albumin; GLB, globulin; FBG, fasting blood glucose; sCr, serum creatinine; UA, uric acid; eGFR, estimated glomerular filtration rate; TG, triglyceride; TC, total cholesterol; HDL-C, high-density lipoprotein cholesterol; LDL-C, low-density lipoprotein cholesterol; hsCRP, high-sensitivity C-reactive protein; IgA, immunoglobulin A; IgG, immunoglobulin G; IgM, immunoglobulin M; C3, complement three; C4, complement 4; CH50, total complement activity; U-RBC, urine red blood cells; 24 h U-PRO, 24-hour urine protein; uPCR, urine protein creatinine ratio; kapU, urine immunoglobulin kap; lamU, urine immunoglobulin lam; IgGU, urine immunoglobulin G; β2-Mg, urine β2-microglobulin; ALBU, albuminuria; α1-MU, urine α1 microglobulin; α2-MU, urine α2 macroglobulin; TrfU, urine Transferrin; M, mesangial hypercellularity; E, endocapillary hypercellularity; S, segmental sclerosis; T, tubular atrophy/interstitial fibrosis; C crescents.

### Composition of the gut microbiota

**FIGURE 1:**
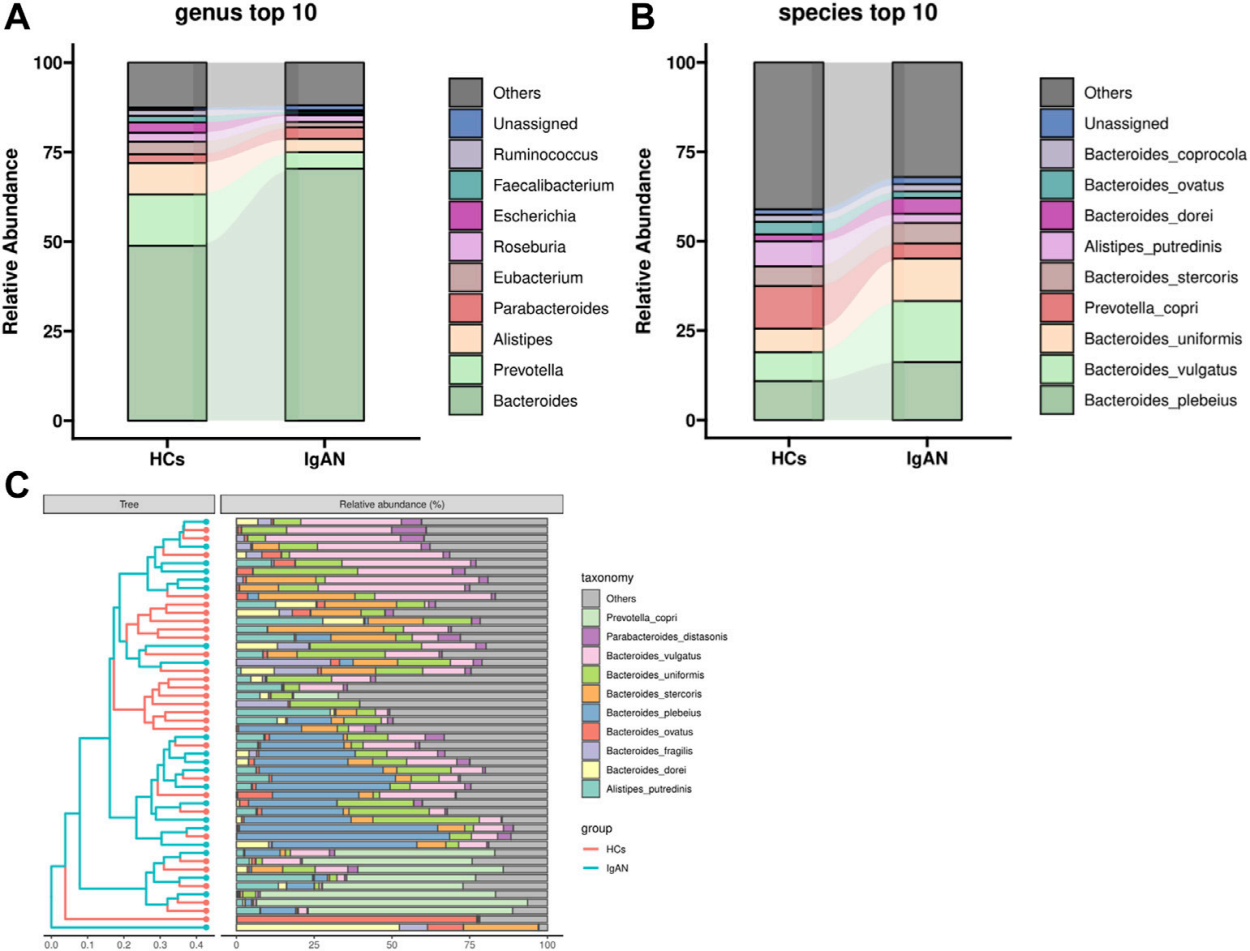
Community Profiling analysis showing differential relative abundances of fecal microbiota in IgAN patients and HCs. **(A)** Microbiome composition of the two groups at the genus level. **(B)** Microbiome composition of the two groups at the species level. **(C)** Relative abundance of the top 10 species in each sample.

Community Profiling analysis showed that there were no significant differences from phylum to species levels between IgAN and HCs groups (Supplementary Figure S1 and Figure 1). Phyla *Bacteroidetes*, *Firmicutes* and *Proteobacteria* predominated among bacteria. Compared to HCs, the level of *Bacteroidetes* was higher in IgAN patients, while *Firmicutes* and *Proteobacteria* were lower. *Bacteroidia* and *Negativicutes* showed an increased abundance in IgAN patients rather than *Clostridia* and *Gammaproteobacteria* in HCs at the class level. At the order level, the IgAN patients showed an increase of *Bacteroidales* and *Burkholderiales* but a decrease of *Clostridiales* and *Enterobacterales*, compared to HCs. In addition to *Bacteroidaceae*, *Prevotellaceae*, *Rikenellaceae* and *Lachnospiraceae*, which are the dominant bacteria at the family level, *Tannerrellaceae* were more abundant in the IgAN group, while the healthy groups were *Ruminococcaceae* and *Eubacteriaceae* (Supplementary Figure S1). At the genus level, *Bacteroides* and *Prevotella* were the most dominant in both groups, in which *Bacteroides* was dominant in the IgAN group, *Prevotella* and *Alistipes* were more abundant in the HCs group (Figure 1A). At the species level, *Bacteroides plebeius* and *Bacteroides vulgatus* were more abundant in the IgAN group, while *Prevotella copri* and *Alistipes putredinis* were more frequent in the HCs group (Figures 1B,C).

**FIGURE 2:**
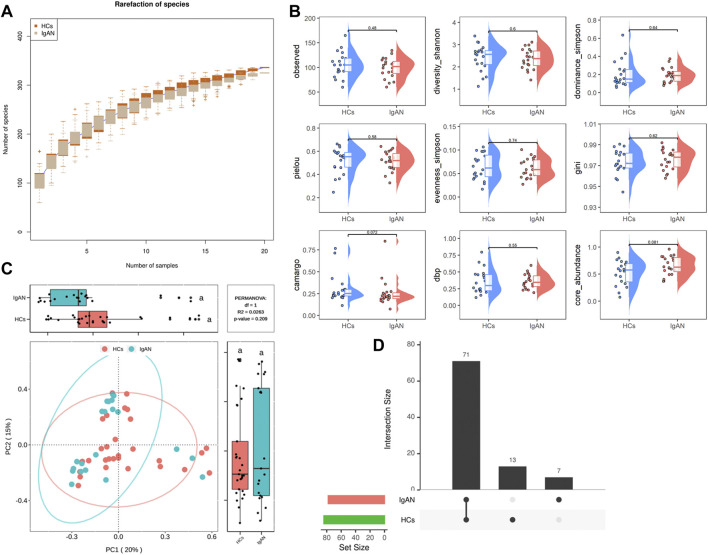
Rarefaction curves and comparison of diversity indexes between IgAN patients and HCs. **(A)** Rarefaction curves of patients with IgAN and HCs at the species level. The sequencing depth was judged to be sufficient as the curve tended to be flat. The detection rate of the microbial community was almost flat, revealing a reasonable sequencing volume that could cover most species. **(B)** α-diversity indexes in IgAN patients and HCs (observed, diversity Shannon and Simpson indexes depict diversity; pielou, evenness Simpson and Gini depict evenness; camargo, dbp and core abundance depict dominance). **(C)** PcoA for β-diversity analysis. Green and red represent different samples from the two groups. The structure and composition of the gut microbiota in patients with IgAN were not significantly different from those of HCs. **(D)** Venn diagram. There were 71 species shared between the two groups, while 13 species were specific to HCs and 7 species were specific to IgAN patients.

Analysis of the rarefaction curves at the species level showed that the dataset in this study was sufficiently large and valid (Figure 2A). To examine differences in the microbial community richness and composition between the two groups, we evaluated the α and β diversity of gut microbiota. In terms of diversity (observed, shannon, simpson), evenness (pielou, simpson, gini), and dominance (camargo, dbp, core), there were no statistically significant differences in the comparison of α-diversity between the two groups, but a separation trend occurred in bacterial community richness between groups. (Figure 2B).

Based on the β-diversity analysis, the PCoA plot showed that there were no significant differences between groups in species composition (*p* = 0.209; Figure 2C), while individual differences were more pronounced in the HCs group (Supplementary Figure S2). Moreover, the Venn diagram analysis showed that a total of 71 species were shared between the two groups, while 13 were specific for HCs and 7 were unique to IgAN (Figure 2D). Overall, these findings indicated that the composition of microbial communities did not differ significantly between groups, but there was a trendency towards variation.

### Differential abundance of intestinal bacteria in IgAN and HCs groups

**FIGURE 3:**
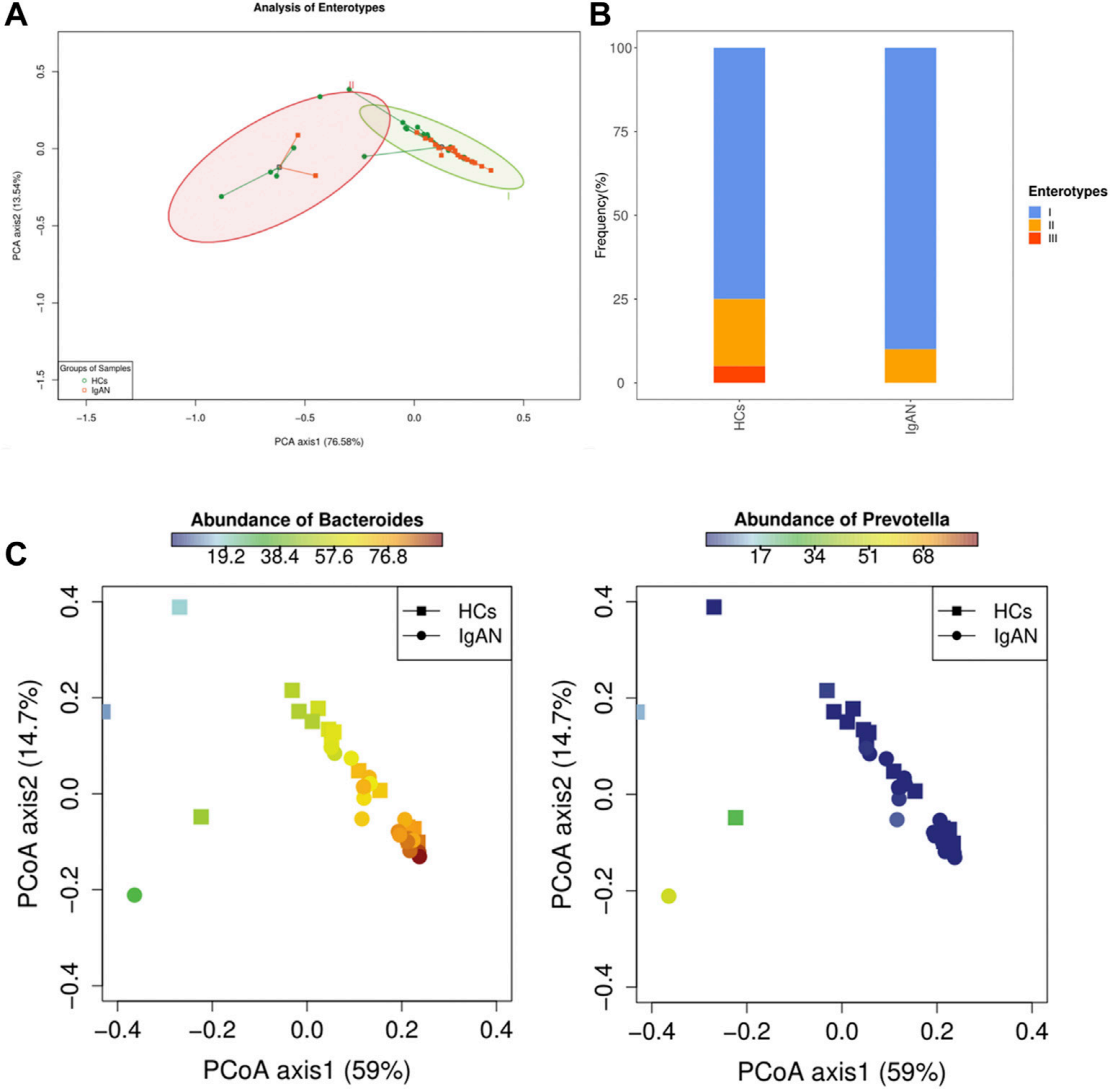
Analysis of Enterotypes between IgAN patients and HCs. **(A)** A PCA plot indicating that enterotypes driven by *Bacteroides* and *Prevotella* were dominants in IgAN and HCs groups, respectively. **(B)** Frequency of enterotypes in both groups. **(C)** Abundance of *Bacteroides* and *Prevotella* between the two groups showed an opposite trend.

To better distinguish the microbiota types of disease and health status, we performed enterotypes analysis. Enterotypes analysis was based on the classification of genus levels, as it better-reflected morphological changes. Enterotypes were usually defined as microbial flora dominated by genus *Bacteroides*, *Prevotella*, or *Ruminococcus* (Arumugam et al., 2011). Samples with a relative abundance of *Bacteroides* (over 40%) and levels greater than *Prevotella* were assigned to enterotype I. Samples with a relative abundance of *Prevotella* (over 30%) and levels greater than or equal to *Bacteroides* were assigned to enterotype II. Samples dominated by *Ruminococcus* were assigned to enterotype III. The weighted PcoA analysis of stool samples demonstrated a strong clustering into two enterotypes dominated by the two genera (Figure 3A). Enterotype I and II contained a high proportion of *Bacteroides* and *Prevotella,* respectively (Figure 3B). The distribution of *Bacterodies* and *Prevotella* showed an opposite trend (Figure 3C). Interestingly, the abundance of enterotype I was similar between HCs and IgAN groups (Supplementary Figure S3), while the frequency of occurrence was higher in the IgAN group (Supplementary Figure S4). These results suggested that the enterotype I driven by *Bacteroides* was strongly associated with IgAN, and the enterotype II driven by *Prevotella* was related to HCs, indicating the differences of intestinal bacteria abundance existed between two groups.

**FIGURE 4:**
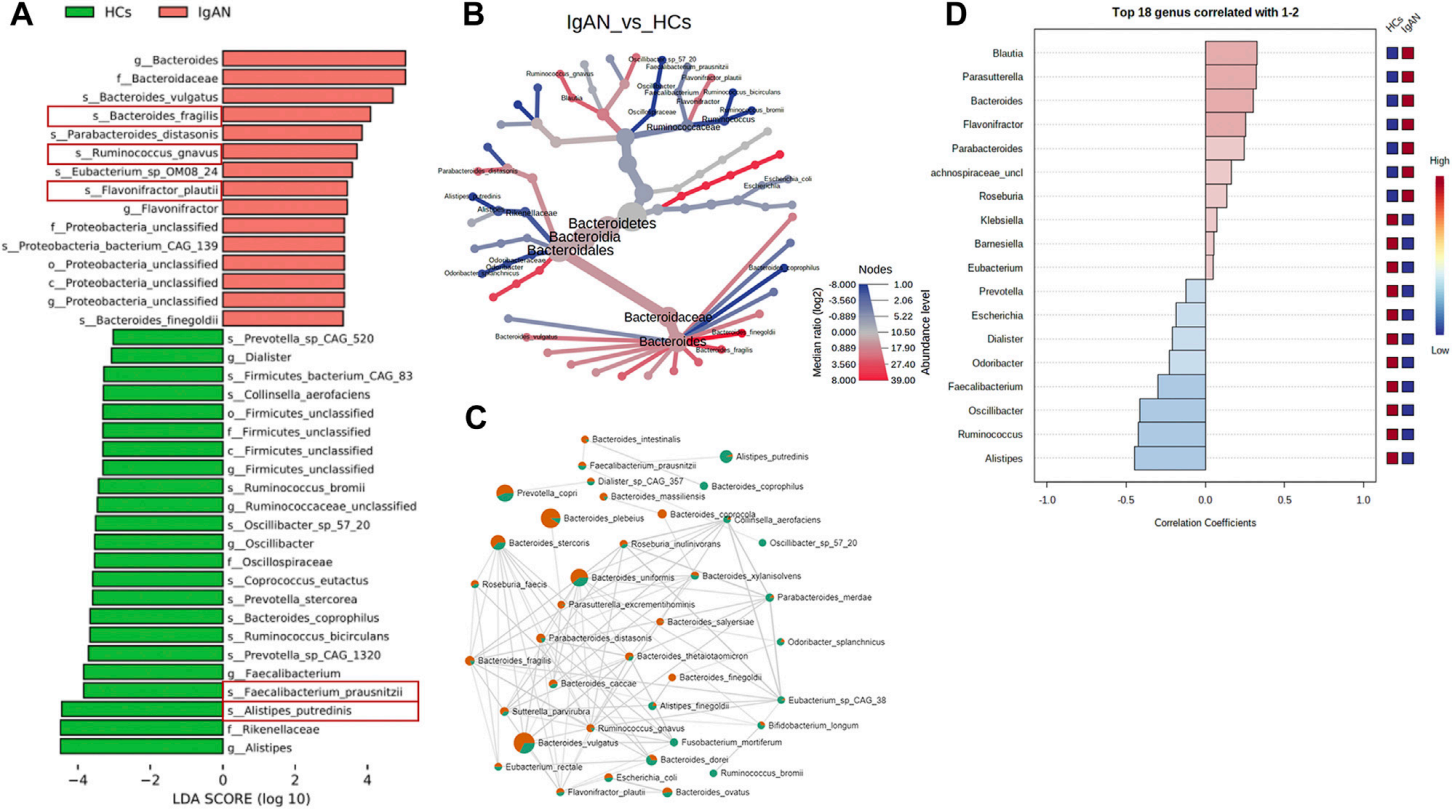
Gut microbiota differences from phylum to species between patients with IgAN and HCs. **(A)** Microbiome biomarkers were identified using a logarithmic linear discriminant analysis (LDA) effect size (LefSe) threshold >4.0. **(B)** HeatTree for clustering analysis. Red indicates a significant increase in abundance in IgAN patients compared to the HCs, while blue depicts the opposite. **(C)** Co-occurrence network analysis of stool microbiota using Pearson’s correlation coefficient. A node represents a species. The node size indicates the level of abundance. Color scale indicates the proportion of bacterium in the two groups separately. Red depicts IgAN patients while green depicts HCs. Connecting lines indicate the strength of the relationship. **(D)** Correlation coefficient rank at the genus level. Red denotes positive associations in IgAN patients, while blue denotes negative associations. The legend on the right indicates the abundance of the bacterium in both groups, with red indicating high and blue indicating low abundance (c = class; o = order; f = family; g = genera; s = species).

To further explore differences in microflora between the groups, we used the LEfSe algorithm to detect the abundance from the phylum to species levels. The results indicated that one class, one order, two families, three genera and eight species were enriched in patients with IgAN, while one class, one order, three families, six genera and 12 species were enriched in HCs (LDA score >2.0, *p* < 0.05) (Figure 4A). At the species level, *Bacteroides fragilis*, *Flavonifractor plautii* and *Ruminococcus gnavus* were characteristic of the IgAN group, whereas *Alistipes putredinis* and *Faecalibacterium prausnitzii* were exclusive to the HCs group (Figure 4A). A cladogram was then generated to visualize and compare the phylogenetic distribution between the two groups from the class to family level. The results supported significant differences presented at each taxonomic level analyzed (*p* < 0.05; Supplementary Figure S5).

The clustering analysis of microbiota enrichment verified the diversity of *Bacteroides* and exhibited clusters for *Alistipes* and *Faecalibacterium* in HCs, which also revealed that IgAN patients had an increased abundance of *Bacteroides finegoldii*, *Bacteroides fragilis* and *Flavonifractor plautii*, compared to HCs (Figure 4B). Moreover, the network map depicting enrichment relationships between groups at the species level illustrated that several bacteria were closely associated with *Bacteroides fragilis*, including *Bacteroides vulgatus* and *Flavonifractor plautii,* among which showed an increase of abundance in IgAN groups and a decrease in HCs (Figure 4C). Alternatively, the Pearson correlation analysis at the genus level found that *Bacteroides* and *Flavonifractor* were positively correlated with IgAN patients along with higher abundance, and found that *Alistipes* and *Ruminococcus* were negatively correlated with IgAN patients, whereas the abundance was higher in HCs group (Figure 4D).

### Correlations between gut microbiota and IgAN clinical characteristics

**FIGURE 5:**
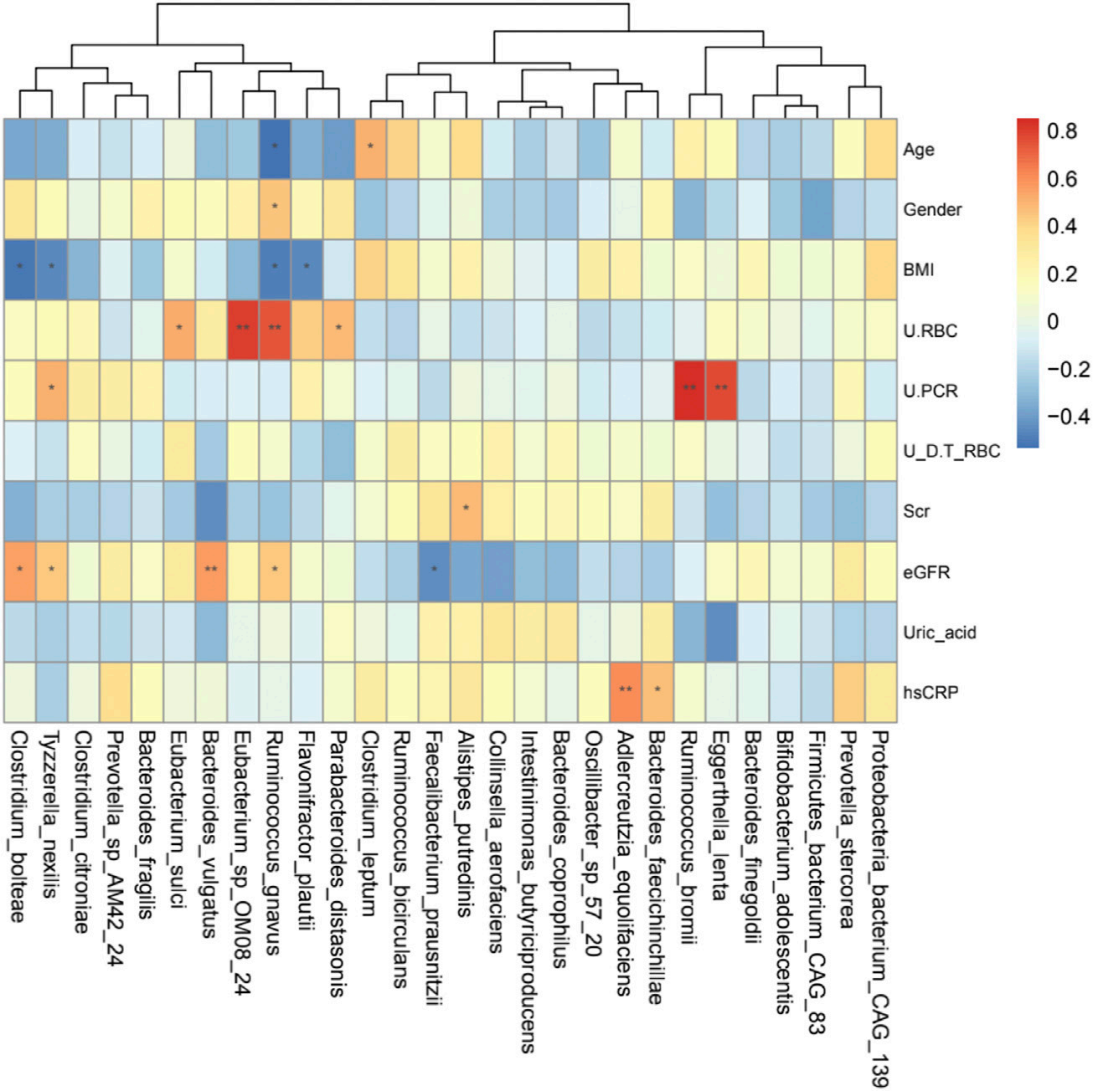
Heatmaps showing correlations between gut microbiota species and IgAN clinical parameters. The intensity of the color indicates the r value (correlation). The red color represents a positive score, and the blue color represents a negative one. *p <0.05 and **p <0.01.

Spearman’s correlation analysis was performed in the IgAN group to explore the relationship between bacteria species and clinical parameters (Figure 5). *Eggerthella lenta* and *Ruminococcus bromii* were positively correlated with urine protein-creatinine ratio. *Bacteroides vulgatus* and *Ruminococcus gnavus* were positively associated with eGFR, and the latter had a direct association with urine red blood cells count. Moreover, *Clostridium bolteae* and *Tyzzerella nexilis* were also positively correlated with eGFR, but negatively correlated with BMI. Besides, *Adlercreutzia equolifaciens* and *Bacteroides faecichinchillae* were positively correlated with hsCRP. These findings illustrated a strong correlation of altered fecal microbiota with clinical and biochemical features of IgAN patients.

### Pathway and function enrichment analysis of differential bacteria

**FIGURE 6:**
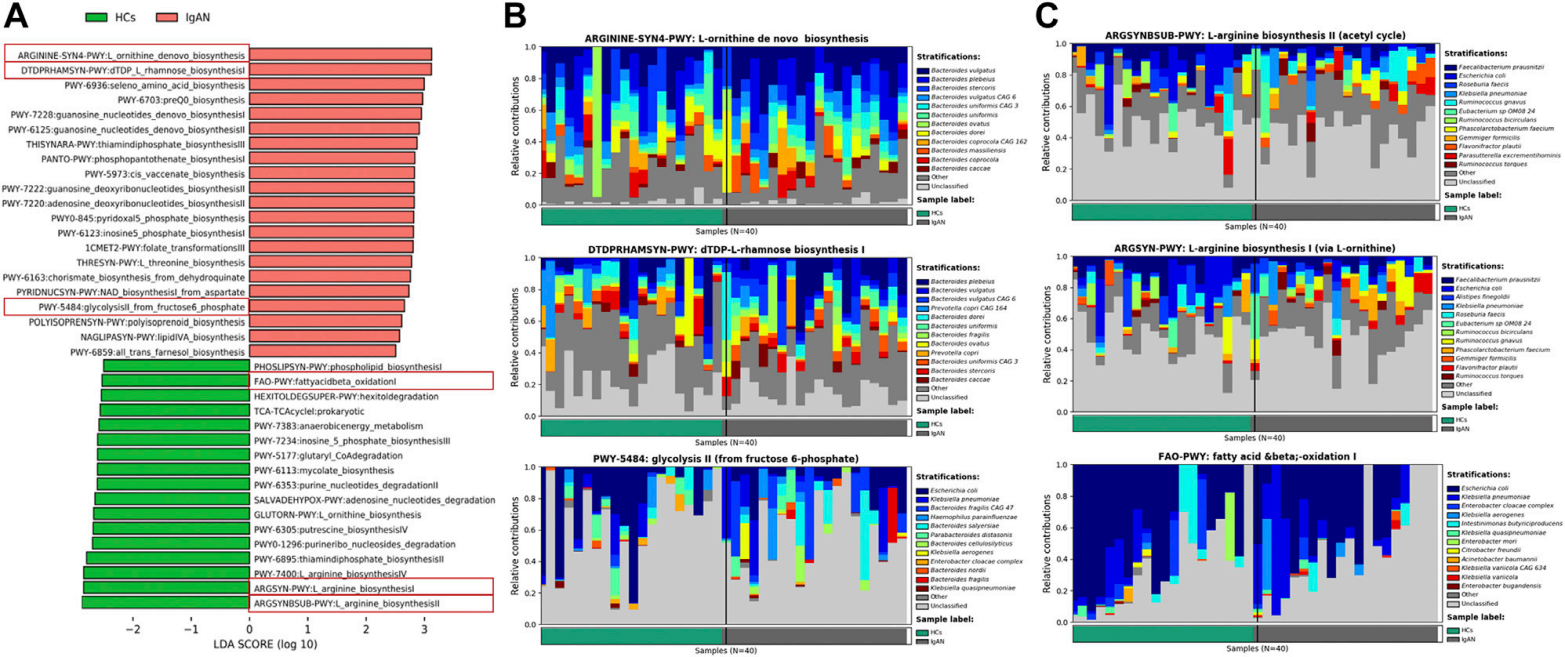
Enrichment of functional pathways of differential bacteria between IgAN patients and HCs. **(A)** The distribution of LDA values for different pathways in IgAN and HCs samples. **(B)** Relative contribution of gut microbes to pathways IgAN patients. **(C)** Relative contribution of gut microbes to pathways in HCs samples. Enrichment was defined as *p* < 0.05, q <0.1 and LDA>3.0.

Based on the MetaCyc database, the LefSe analysis showed the enrichment of pathways in the two groups (Figure 6A). The gut microbiota-related pathways enriched in the IgAN group were involved in the biosynthesis of amino acids and glycans, of which most of the contributing strains were derived from *Bacteroides* (Figure 6B). Meanwhile, dominant pathways for the HCs group included the biosynthesis of amino acids and fatty acid, especially the biosynthesis of L-arginine, originating from various genus such as *Faecalibacterium*, *Escherichia* and *Ruminococcus* (Figure 6C). The MetaCyc pathway analysis predicted the different metabolic functions of fecal microbiota between HCs and IgAN.

### Enzymes associated with specific bacteria were enriched in IgAN

**FIGURE 7:**
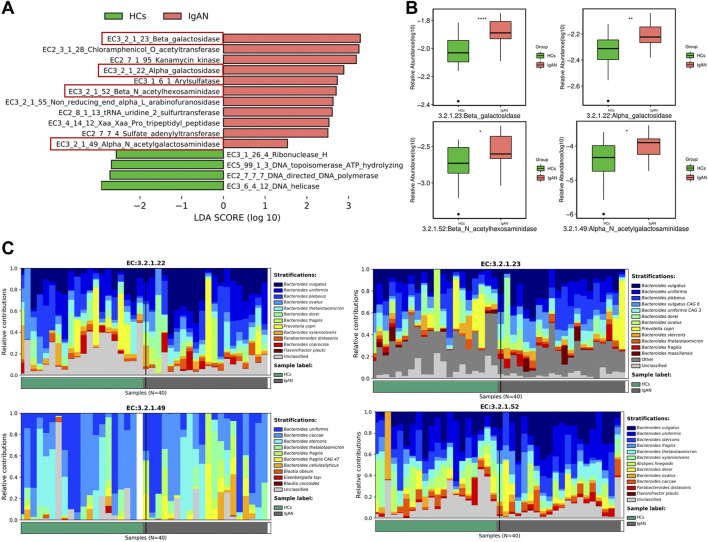
The enrichment of active enzymes and their corresponding specific bacteria. **(A)** Enzymes enriched by gut flora in IgAN patients and HCs. **(B)** The relative abundance of β-galactosidase, β-N-acetylhexosaminidase, α-galactosidase and α-N-acetylgalactosaminidase between the IgAN group and HCs. **(C)** The stratification of various bacteria for β-galactosidase, β-N-acetylhexosaminidase, α-galactosidase and α-N-acetylgalactosaminidase.

Gut microbiota produces dozens of enzymes to influence host physiology and human disease. Therefore, we identified enzymes enriched in IgAN and HCs groups based on the functional analysis of metagenomics data. We found that β-galactosidase (EC 3.2.1.23), β-N-acetylhexosaminidase (EC 3.2.1.52), α-galactosidase (EC 3.2.1.22) and α-N-acetylgalactosaminidase (EC 3.2.1.49) were enriched in IgAN samples (Figure 7A). Indeed, when compared to the HCs group, β-galactosidase, β-N-acetylhexosaminidase, α-galactosidase and α-N-acetylgalactosaminidase were significantly enriched in the IgAN group (Figure 7B). Interestingly, β-galactosidase helps to degrade milk lactose in the process of generating lactose-free milk (Saqib et al., 2017). A recent study suggested that A/B antigens influence the prognosis of IgAN patients through the association of O-glycan structures in the IgA1 hinge region, which indicated an association between ABO blood group and Gd-IgA1 (Wang et al., 2021). Another research found that α-galactosidase and α-N-acetyl-galactosaminidase from *Flavonifractor plautii*, could remove the GalNAc or Gal structures from A/B antigens, as a way of converting A or B RBCs to O (Rahfeld et al., 2019). Therefore, α-galactosidase and α-N-acetylgalactosaminidase were probably implicated in the pathogenesis of IgAN since they are key to protein glycosylation. In terms of the relative contribution of gut microbiota, these results indicated that the genus *Bacteroides* and species *Flavonifractor plautii* exerted the most important influence on enzyme production (Figure 7C), which indicated that specific bacteria and enzymes might contribute to the pathogenesis of IgAN.

## Discussion

This was a retrospective study that investigated the fecal microbiota diversity and function in Chinese IgAN patients through shotgun metagenomics sequencing. The results showed a degree of differences in the richness and composition of gut microbiota between IgAN and HCs groups. The enterotype I driven by *Bacteroides* was closely associated with IgAN. Moreover, *Bacteroides fragilis*, *Flavonifractor plautii* and *Ruminococcus gnavus* were characteristic bacteria enriched in IgAN that showed marked correlations with clinical features. Besides, microbiota-related amino acids and glycans biosynthetic pathways as well as inflammation pathways were enriched in IgAN samples. Furthermore, enzyme analysis corroborated that Gd-IgA1-associated α-galactosidase and α-N-acetylgalactosaminidase from *Flavonifractor plautii* were enriched in the IgAN group.

It is well known that dietary habit plays a key role in shaping gut microbiome in the human, of which short- or long-term dietary changes could influence the ecology of the gut microbiome (Costea et al., 2018). To avoid the effect of different dietary patterns on gut microbiome and relevant disease, this study first evaluated the dietary habits between IgA patients and Healthy controls. These results indicated that there were no significant differences in lifestyle and dietary habits between the two groups. Based on the previous studies on IgA nephropathy (Hu et al., 2020; Zhong et al., 2020), there are generally not detailed nutritional data, such as carbohydrate, protein, and calorie intake, when evaluating the effect of dietary habits on nutrition intake. We speculate that the dietary habits between IgAN patients and healthy controls are roughly similar. Another possibility is that it is not easy to collect nutrition information among IgAN patients in clinical practice. Thus, we just analyzed dietary and lifestyle information and didn’t further assess the nutritional state.

Metagenomics sequencing results indicated that the diversity of the fecal microbiota from phylum to genus levels partially differed between IgAN patients and controls (Supplementary Figure S1–4; Figures 1A,B). The community richness and composition of the gut microbiome were lower in IgAN patients when compared to that of HCs. This agrees with previous observations using 16S rRNA sequencing (Hu et al., 2020; Zhong et al., 2020). At the genus levels, we found that *Bacteroides* was dominant in patients with IgAN, while *Prevotella* and *Alistipes* were more abundant in HCs (Figure 1A). *Prevotella* and *Alistipes* are considered to be beneficial for human physiology, having potential anti-inflammatory effects (Parker et al., 2020; Tett et al., 2021). As for *Bacteroides*, we showed that *Bacteroides* contributed the most in differentiating IgAN from controls (Figure 4B). Indeed, *Bacteroides* was shown to impact fecal IgA levels (Yang et al., 2020). Depending on their locations in the host, some species of *Bacteroides* may exert a beneficial or pathogenic effect, in which gut *Bacteroides* is usually perceived as having a beneficial effect on health, while *Bacteroides* found in other body locations are perceived as opportunistic pathogens (Zafar and Saier, 2021).

Compared to 16S rRNA sequencing, which can only analysis to genus level, metagenomics sequencing can accurately perform the corresponding annotation at the species level. From the community composition, *Bacteroides plebeius* and *Bacteroides vulgatus* were higher in IgAN, whereas *Prevotella copri* and *Alistipes putredinis* showed a higher abundance in HCs (Figure 1B). *Bacteroides vulgatus* was shown to be increased in patients with active intestinal bowel disease and exacerbated colitis by modulating NF-κB (Ó Cuív et al., 2017), while *Prevotella copri* is potentially beneficial in glucose homeostasis and host metabolism (Asnicar et al., 2021). More importantly, differential bacteria in both groups were found by LefSe, which indicated that *Bacteroides fragilis*, *Flavonifractor plautii* and *Ruminococcus gnavus* were characteristic species of IgAN, whereas *Alistipes putredinis* and *Faecalibacterium prausnitzii* were exclusive to HCs (Figure 4A). Some studies have illustrated that IgA secreted by the host in the gut, can bind mucus and strengthen the mucosal colonization of *Bacteroides fragilis* (Sun et al., 2019). A case report proposed that a bloodstream infection of *Flavonifractor plautii* occured after infectious colitis, which suggested that damages to the intestinal wall favored the transmission of this bacteria (Karpat et al., 2021). *Ruminococcus gnavus* was shown to be associated with Crohn’s disease because it produces an inflammatory polysaccharide (Henke et al., 2019). On the other side, *Alistipes putredinis* and *Faecalibacterium prausnitzii* were reported to exert anti-inflammatory effects (Lenoir et al., 2020; Parker et al., 2020). Our findings also suggested a correlation between intestinal microflora and clinical characteristics of IgAN patients. Spearman correlation analysis **(** Figure 5 **)** confirmed that *Clostridium bolteae*, *Tyzzerella nexilis*, *Bacteroides vulgatus* and *Ruminococcus gnavus*, were positively correlated with eGFR, while *Eggerthella lenta* and *Ruminococcus bromii* were positively correlated with the urine protein-creatinine ratio, both of which are markers of renal damage. *Ruminococcus gnavus* showed a direct association with urine red blood cell count, which was elevated to the diagnosis of hematuria. It is known that hematuria is a typical symptom in patients with IgAN. Therefore, changes in the gut microbiome could lead to changes in relevant clinical parameters, suggesting that the altered gut microbiota might be potential biomarkers for IgA nephropathy.

Our present study was the first to characterize the interaction between enterotypes and IgAN using metagenomics sequencing. An analysis of gut microbial communities proposed three predominant “enterotypes” dominated by *Bacteroides, Prevotella,* and *Ruminococcus*, respectively (Arumugam et al., 2011). Indeed, enterotypes, or their main taxonomic drivers, are associated with human diseases (Costea et al., 2018). Two enterotypes were found in our study based on the abundance of microbial genera. The enterotype driven by *Bacteroides* was dominant in our samples and *Bacteroides* was enriched in patients with IgAN. An increase of *Bacteroides* or enterotype I itself, which tends toward a lower overall diversity, has been linked to colorectal cancer, celiac disease, immune-senescence, and constant low-grade inflammation (Costea et al., 2018), which can also influence antibody reactivity and Th cell subsets (Grosserichter-Wagener et al., 2019). It was suggested that enterotype I-derived microbes aid in the fermentation of carbohydrates and proteins, because they are enriched in genes representing saccharolytic enzymes, galactosidases, hexosaminidases, proteases, and enzymes in the glycolysis and pentose phosphate pathways (Arumugam et al., 2011). A recent cross-sectional study showed that long-term diet is strongly associated with enterotypes partitioning (Wu et al., 2011). There should be a degree of correlation of dietary habits, enterotypes, and disease. Interestingly, carbohydrates are the staple food in Asia, and dietary habits in our study reinforced this and were not different between groups (Table.1). Moreover, IgAN in Asian regions accounts for 45.3% of primary glomerulonephritis cases (Li and Liu, 2004). Thus, the relationship between enterotypes and IgAN should be further explored.

Pathways enriched in IgAN samples were identified (Figures 6A–C). Pathway analysis based on MetaCyc demonstrated that the gut microbiota of patients with IgAN was enriched in multiple biosynthesis pathways, especially the biosynthesis of glycans and amino acids. It is known that glycans are involved in the formation of normal IgA1 structures, while aberrantly glycosylated IgA1 in patients with IgAN is associated with galactose-deficient O-glycans. Thus, the enrichment of the biosynthesis pathway of glycans might related to the changes of normal IgA1, leading to IgAN. Besides, the amino acid metabolism mediated by gut microbiota plays an important role in CKD. Abnormal amino acid metabolic pathway enrichment caused by gut microbiota may play an important role in kidney damage (Feng et al., 2019; Ren et al., 2020), leading to the progression of IgAN. Moreover, most of the contributing strains for those pathways were based on the genus *Bacteroide*, suggesting that *Bacteroides* may be involved in the progression of IgAN.

Metagenomics was used to detect enzymes enriched in feces that could reflect the functional exertion of intestinal bacteria, which is difficult for 16S rRNA sequencing to achieve. Interestingly, the relative abundances of α-galactosidase and α-N-acetylgalactosaminidase were higher in IgAN than those were in HCs, suggesting that these enzymes may influence the pathogenesis of IgAN. In addition, these two enzymes were not only enriched in *Bacteroides* but also in *Flavonifractor plautii*. Notably, a recent study identified *Flavonifractor plautii* a key bacterium associated with colorectal cancer in Indian patients (Gupta et al., 2019). Indeed, α-galactosidase and α-N-acetyl-galactosaminidase are enzyme pairs from *Flavonifractor plautii* that work in concert to cleave the A and B antigens (Rahfeld et al., 2019), possibly exerting a similar effect in the production of Gd-IgA1 since Gd-IgA1 is affected by modifications of N-acetylgalactosamine and galactose in IgAN patients. Thus, the subsequent analysis particularly focused on *Flavonifractor plautii* rather than *Bacteroides* in IgAN patients.

Gd-IgA1 is regarded as an important effector molecule in IgAN (Suzuki et al., 2018). However, the exact production site of Gd-IgA1 remains controversial. Recent research suggested that A/B antigens influence the prognosis of IgAN patients through the association of O-glycan structures in the IgA1 hinge region, and the finding indicated an association between ABO blood group and Gd-IgA1 (Wang et al., 2021). Moreover, red blood cell surface antigens are carbohydrate structures that respectively terminate in GalNAc or Gal for A and B blood types (Rahfeld et al., 2019). Additionally, it was reported that α-galactosidases can cleave the B antigen substrate (α-Gal), while the combination of α-N-acetylgalactosaminidases and α-galactosidases can convert A^+^ RBCs to O-type RBCs. Both enzymes could be secreted by *Flavonifractor plautii* (Rahfeld et al., 2019). Besides, the identification of O-glycans in normal IgA1 is due to the presence of GalNAc with a β1,3-linked Gal attached by sialic acid (Ohyama et al., 2021). Despite differences in the form of saccharide chains and enzymes involved in glycosylation between ABO and O-glycosylation of IgA1, antigen A and Gd-IgA1 have GalNAc as their terminal oligosaccharide, whereas antigen B and IgA1 present the same terminal oligosaccharide Gal. Here we reported that stool samples from IgAN patients were enriched in α-galactosidase and α-N-acetylgalactosaminidase, which were mainly secreted by *Flavonifractor plautii.* It is known that IgA is secreted into the intestinal lumen and adheres to the mucosa, which mediates microbial homeostasis (Huus et al., 2021). Moreover, the gut microbiota utilizes IgA for mucosal colonization to exert an important role in host physiopathological processes (Donaldson et al., 2018). In conjunction with previous studies, we believe that the altered enzymatic response caused by the imbalance of intestinal bacteria in IgAN patients may be an important factor to trigger the production of Gd-IgA1. Nevertheless, this hypothesis requires further experimental confirmation.

Several limitations should be noted in our study. First, we could not investigate the microbial differences at the different stages of IgAN due to the retrospective study design. A longitudinal study is therefore needed to address this question. Second, the sample size was relatively limited thus a large-scale study involving different populations is needed to confirm the present findings. Moreover, the levels of Gd-IgA1 in serum should be examined to further clarify its association with the specific microbiota enzymes among IgAN patients. Finally, it is necessary to develop experimental studies to validate the hypotheses herein proposed.

## Conclusion

Overall, this study demonstrated that intestinal dysbacteriosis occurred in IgAN patients and was associated with clinical and biochemical features such as eGFR, hematuria and urine protein. These findings suggested that α-galactosidase and α-N-acetylgalactosaminidase secreted by *Flavonifractor plautii* might be related to the production of Gd-IgA1 in IgAN patients, indicating that certain enzymes originated from abnormal intestinal microbiota may contribute to the production of Gd-IgA1 and influence the development of IgAN. Results herein reported shed new light on the pathogenesis of IgAN.

## Data Availability

The datasets presented in this study can be found in online repositories. The names of the repository/repositories and accession number(s) can be found in the article/Supplementary Material.

## Supplementary material

Supplemetary Figure S1 Microbiome composition of the two groups from the phylum to family level.

Supplemetary Figure S2 Adosim analysis between the IgAN group and HCs (P=0.691).

Supplemetary Figure S3 The difference of abundance of enterotye I between two groups.

Supplemetary Figure S4 The difference of frequency of enterotye I between two groups.

Supplemetary Figure S5 Cladograms of bacterial lineages with obvious differences between two groups at various levels from class to family. Six bacterial taxa were significantly enriched in patients with IgAN (red) while ten were significantly enriched in HCs (blue).
