# Supplementary figures and images for: Metagenomics-based systematic analysis reveals that gut microbiota Gd-IgA1-associated enzymes may play a key role in IgA nephropathy
Source: Front Mol Biosci. 2022 Aug 24;9:970723. doi: 10.3389/fmolb.2022.970723 (PMC9449366; doi:10.3389/fmolb.2022.970723)

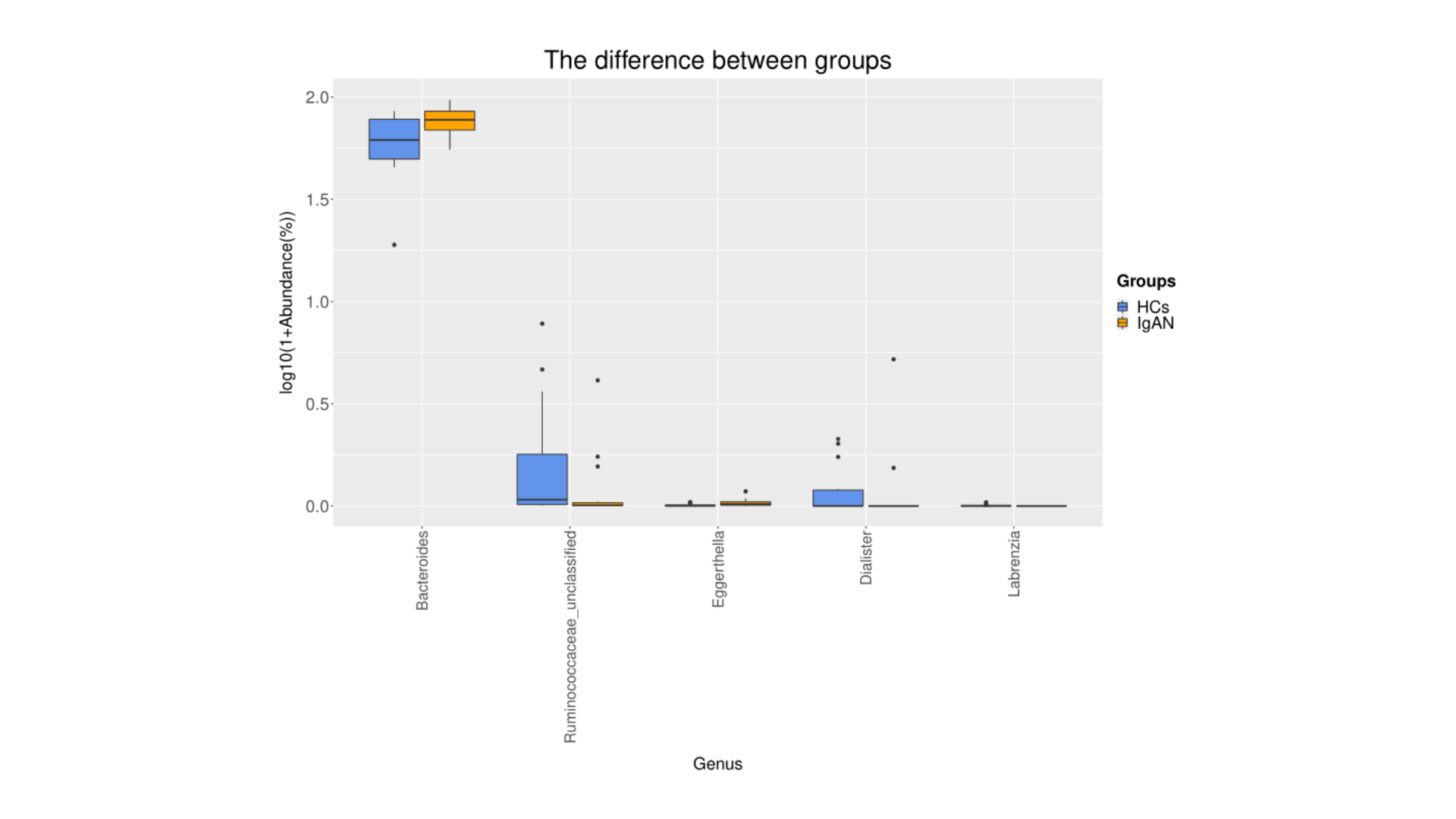

Supplement: Supplementary file 2 [file Image3.TIF]

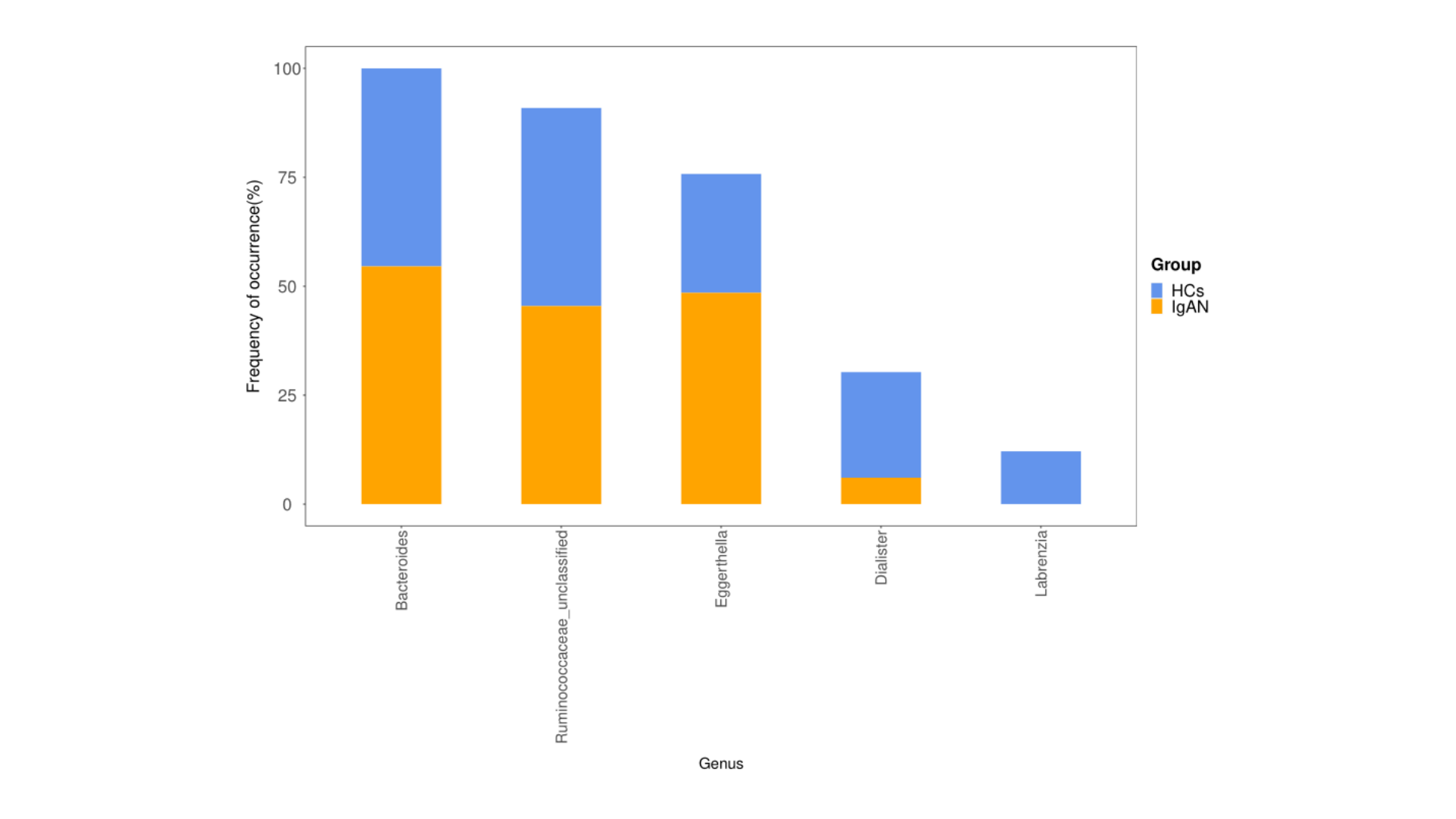

Supplement: Supplementary file 3 [file Image4.TIF]

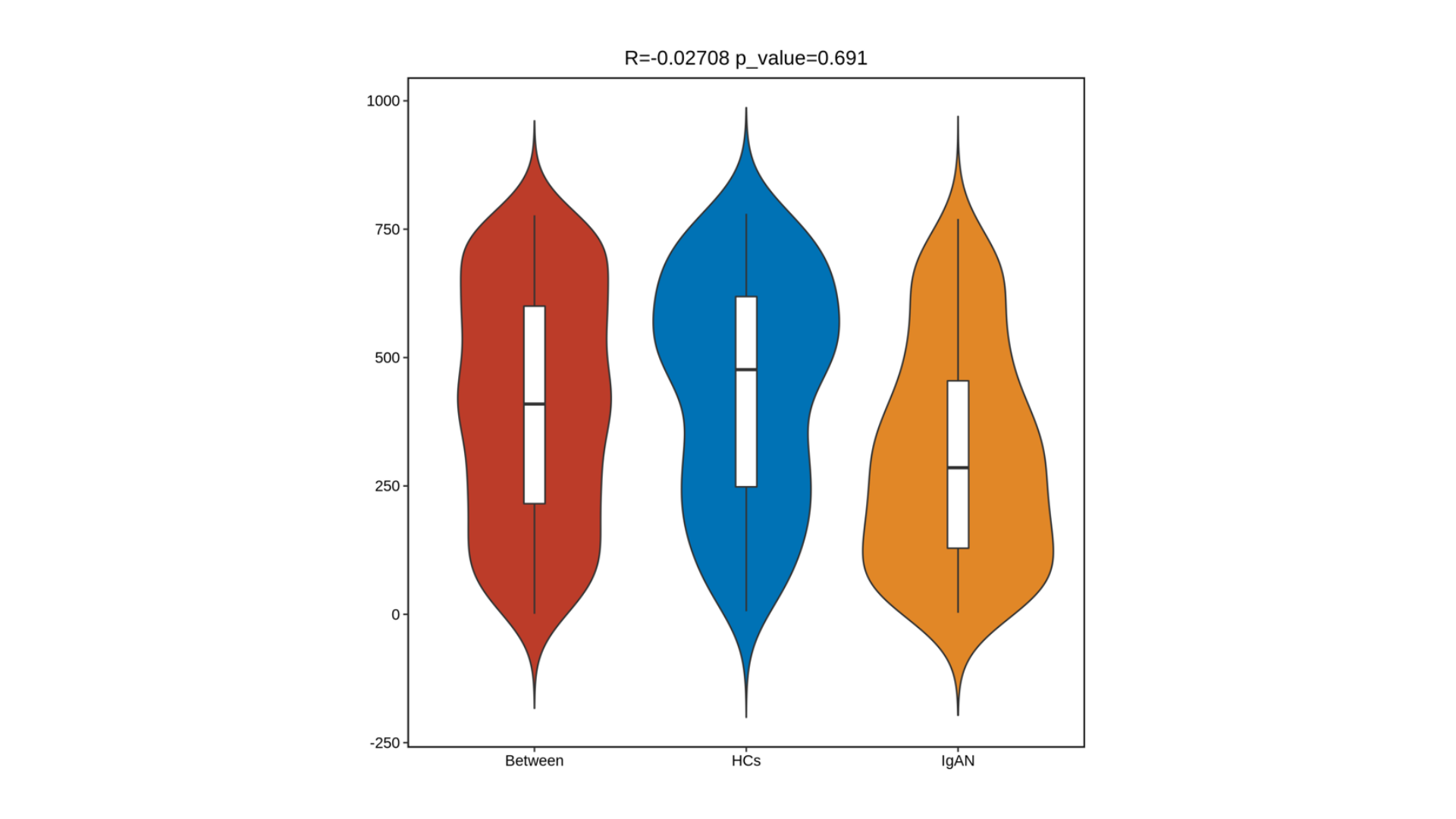

Supplement: Supplementary file 4 [file Image2.TIF]

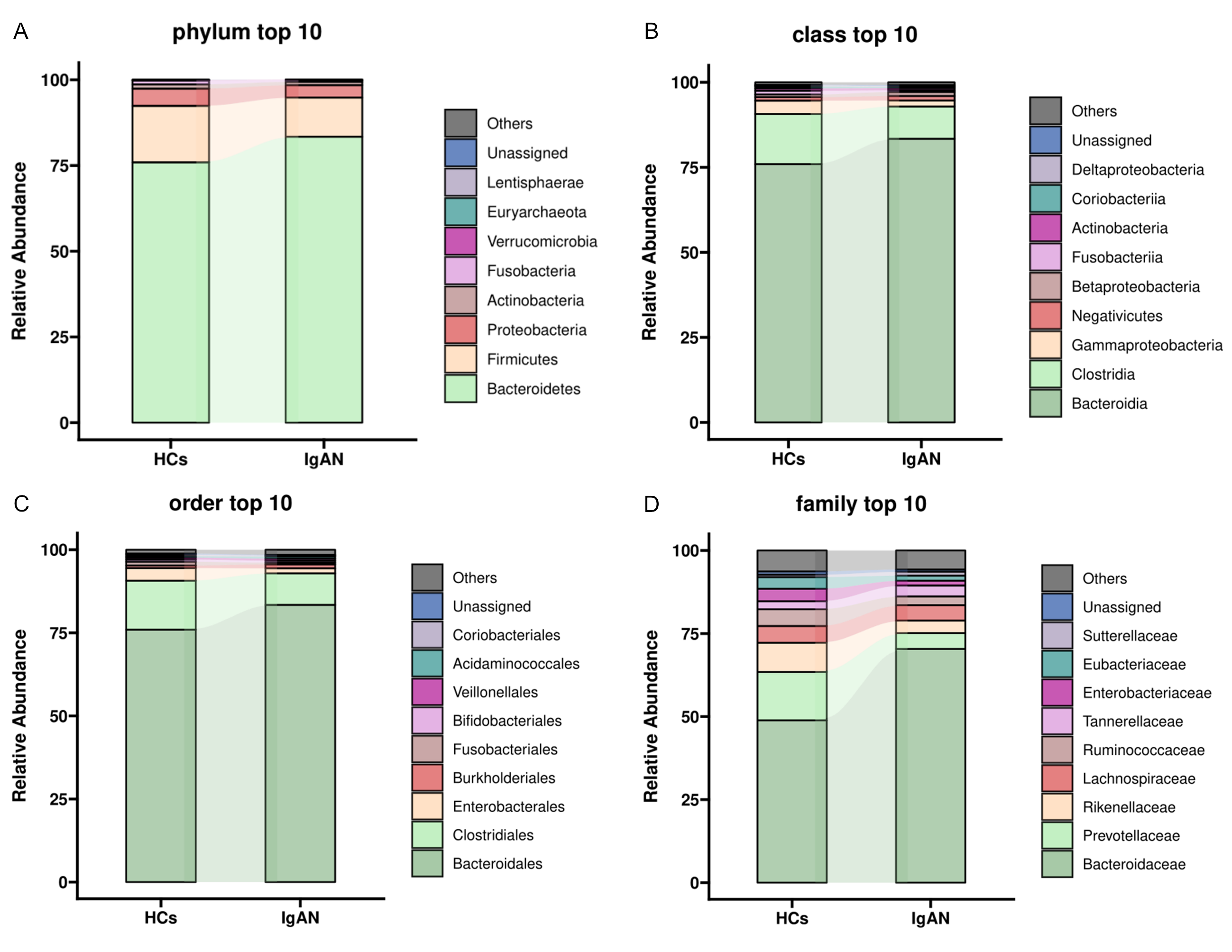

Supplement: Supplementary file 5 [file Image1.TIF]

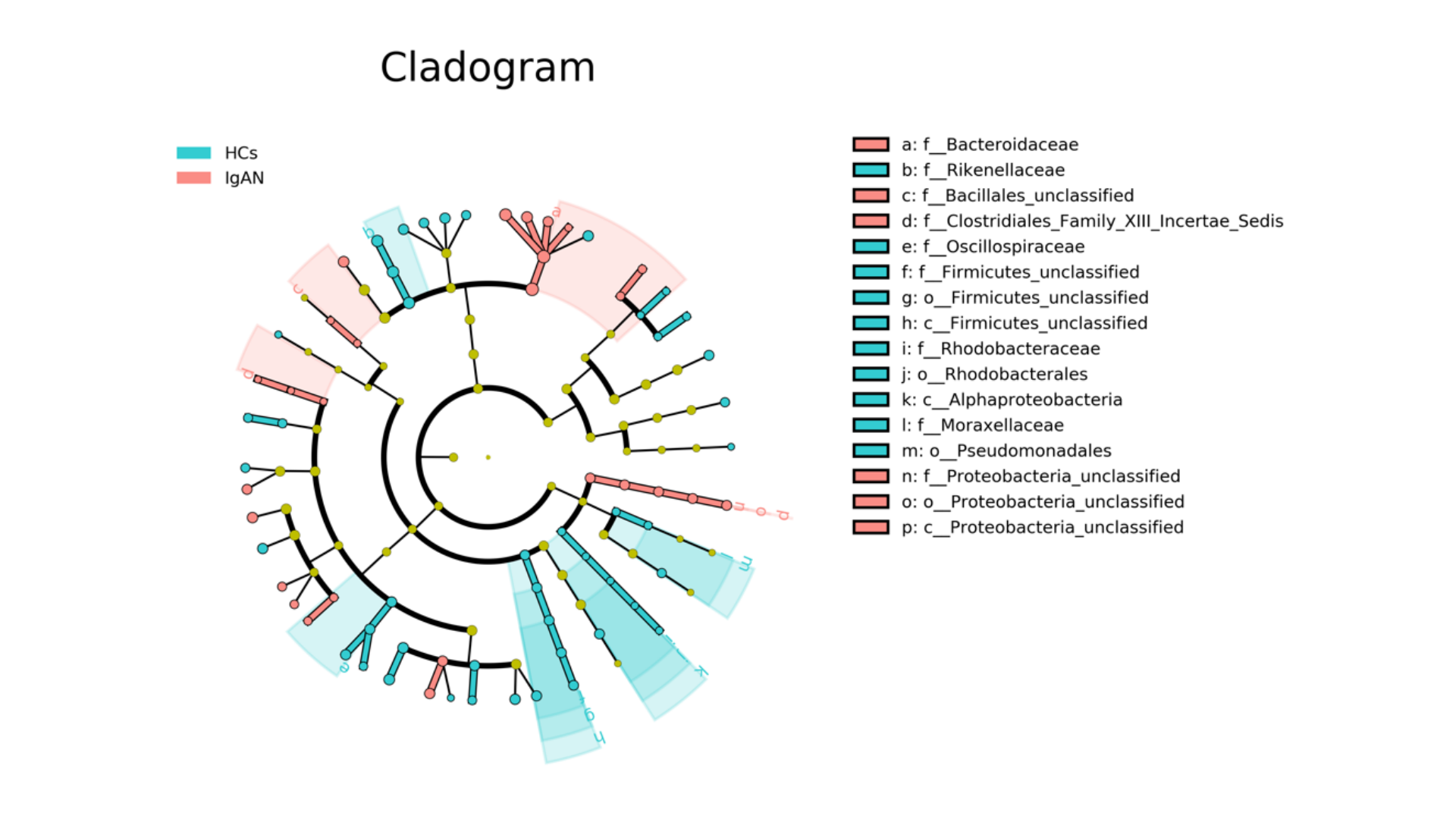

Supplement: Supplementary file 7 [file Image5.TIF]
